# Preterm Birth Prevention in Appalachian Kentucky: Understanding Barriers and Facilitators Related to Transvaginal Ultrasound Cervical Length Surveillance Among Prenatal Care Providers

**DOI:** 10.1089/whr.2019.0023

**Published:** 2020-08-24

**Authors:** Anna Hansen, Mairead E. Moloney, Cynthia Cockerham-Morris, Jing Li, Niraj R. Chavan

**Affiliations:** ^1^ University of Kentucky College of Medicine, Lexington, Kentucky, USA.; ^2^ Department of Sociology, University of Kentucky College of Arts and Sciences, Lexington, Kentucky, USA.; ^3^ Division of Maternal Fetal Medicine, Department of Obstetrics & Gynecology, University of Kentucky College of Medicine, Lexington, Kentucky, USA.; ^4^ Center for Health Services Research (CHSR), University of Kentucky College of Medicine, Lexington, Kentucky, USA.

**Keywords:** preterm birth, cervical length measurement, rural obstetric care, prenatal care providers

## Abstract

***Background:*** Appalachian Kentucky has higher-than-average rates of preterm birth (PTB)—a health disparity associated with increased maternal and fetal/neonatal morbidity and neonatal mortality. Transvaginal ultrasound (TVU) cervical length measurement is the best predictor of PTB risk, but is underutilized in Appalachia. This study explores prenatal care providers' TVU-related knowledge and practices, and identifies barriers and facilitators, which impact the adoption of this evidence-based technology.

***Materials and Methods:*** This study recruited providers from three Appalachian Kentucky health care sites. Prenatal care providers took part in semistructured interviews and completed brief survey scales. Questions focused on PTB knowledge, TVU-related barriers, and suggestions for clinician and/or patient-focused interventions. Transcripts were coded using a multistage process based in grounded theory. Descriptive statistics were calculated.

***Results:*** Eleven physicians, one nurse practitioner, one physician assistant, and one midwife completed interviews. Average participant age was 44 years with 17 years in practice; 43% of providers were female. Practitioners described the sociodemographic characteristics, health behaviors (*e.g.*, smoking, opioid abuse), and comorbid conditions (*e.g.*, obesity, hypertension, and diabetes) endemic in Appalachia that heightened their patients' PTB risk. TVU use was reported as important by all respondents, but not all were satisfied with their level of training. The most commonly identified barriers to TVU were patient access to transportation and social support. Participants stressed a need for changing community perceptions regarding consequences of PTB.

***Conclusions:*** Providers identified multiple TVU-related barriers and facilitators. These data will inform the design of a multifaceted dissemination and implementation strategy targeting PTB prevention in Appalachia.

## Background

According to Healthy People 2020, preterm birth (PTB) is one of the most pressing issues for modern maternal/child health in the United States.^1^ PTB before 37 weeks of gestation is associated with increased maternal morbidity and places neonates at higher risk of early death or disability.^2^ PTB prevention is associated with a significant cost savings associated with reduced lifelong disability and decreased length of stay in the NICU for neonates, and is a prime objective in the reduction of national health disparities.^3–5^

Transvaginal ultrasound (TVU) measurement of cervical length has been demonstrated as the most sensitive biomarker for early PTB in large prospective studies.^6,7^ Unlike transabdominal ultrasound, TVU is not affected by maternal obesity, position of the cervix, and shadowing from the fetus, minimizing interobserver variation.^8–12^ Institutions that have implemented cervical length screening programs have reported a reduction in the rate of PTB and NICU utilization,^13–16^ and universal screening is considered a cost-effective intervention for PTB prevention.^17^ Despite previous research indicating that a universal TVU screening program is acceptable to the vast majority of women,^15^ TVU screening for cervical length is far from universal and uptake rates vary by institution and geographic region.^18^

To promote maternal/child health and reduce financial burden resulting from PTB, barriers to TVU cervical length screening must be identified. Previous work has identified education, awareness, and logistical complications on the part of both patient and provider as barriers for implementation of TVU screening.^19^ Notably, the study of TVU and PTB disparities has focused on racial and global discrepancies.^20,21^ Scant data exist concerning barriers that pertain to rural, health disparate populations where providers—and their resources—may be limited. In addition, past studies have relied on quantitative analyses, particularly *via* survey data.^18,22^

This study uses a mixed-method approach to explore attitudes toward PTB prevention and TVU utilization within a rural, health disparate population. Specifically, semistructured interviews and survey scales assess prenatal providers' TVU-related knowledge and practices, and identify barriers and facilitators that impact the adoption of this evidence-based technology in Appalachian Kentucky. Appalachian Kentucky has PTB rates more than 1.5 times that of the state overall, and almost twice as much as the national average.^23,24^ No known previous research has offered insight into the factors fueling higher-than-average rates of PTB and the use—or lack thereof—of TVU screening for cervical length in this region.

There is a shortage of health care providers in Appalachia,^25^ and prenatal providers may be the sole provider with whom a patient interacts. Prenatal providers in the region represent varying levels of training and diverse skill sets in delivering prenatal care, and may include physicians (*e.g.*, obstetrics and gynecology, family medicine), nurse practitioners, and nurse midwives. Further complicating the clinical picture, providers in Appalachian Kentucky may have reduced access to resources, including technology, and serve a relatively homogenous yet medically complex patient population.^26^

Due to high rates of comorbid conditions, premature death, and their underserved rural status, Appalachian women meet the NIH criteria for a health disparate population, and this has troubling implications for women of childbearing age.^27,28^ Appalachian women exhibit higher rates of comorbid conditions and health behaviors associated with PTB compared with women nationally, including higher-than-average rates of diabetes, hypertension, obesity, and smoking.^27,28^ Moreover, Appalachian women face barriers to care, including lack of transportation, low health literacy, and limited resources required for increased frequency of health care visits.

Appalachian Kentucky's high rates of PTB, limited health care access, and complex patient population demonstrate a strong need for assessing the uptake of TVU cervical length surveillance as an intervention for PTB prevention. Results of this study will ultimately be used in the creation of a customized implementation strategy targeting PTB prevention in Appalachian Kentucky. This study triangulates outcomes of interest by collecting data from both the aforementioned rural obstetric care providers and a subsample of their patient population who have experienced a PTB. The present article describes providers' perspectives.

## Materials and Methods

This study utilized semistructured interviews in conjunction with brief survey scales to elicit insight into prenatal care providers' knowledge, attitudes, and practices with regard to TVU utilization and PTB prevention. To provide further insight into TVU uptake, medical record data from community-based sites were reviewed to determine rates of TVU cervical length measurement among pregnant patients with histories of prior PTBs. The study was approved by the Institutional Review Board at the University of Kentucky.

### Interview participants

Prenatal care providers were recruited from three community-based Appalachian health care sites in Hazard, Morehead, and Ashland, Kentucky. The sites were identified from among the existing community-based affiliates of the Kentucky Angels Telemedicine and Perinatal Outreach program, which represents an existing clinical affiliation for perinatal outreach between the University of Kentucky Division of Maternal Fetal Medicine and the chosen community-based sites. Sites were selected in counties with some of the highest PTB incidence in Kentucky and the United States.^24^ Providers at obstetric practices within the highlighted counties were identified and then approached about participating in the study. Prenatal care providers included a mix of practitioners with subspecialty training in obstetrics and gynecology and family medicine, as well as nurse midwives, nurse practitioners, and physician assistants who functioned as independent prenatal care and obstetric delivery providers. Each site typically records an annual volume of 250–500 deliveries and is staffed by three to five providers, including both physicians and nonphysicians. For instance, prenatal care providers at one of the Appalachian community-based sites include two obstetricians, one family medicine physician, and one physician assistant. Prenatal care providers at a second site include three obstetricians and one nurse midwife.

### Data collection

Guided by the Consolidated Framework for Implementation Research (CFIR),^29^ semistructured qualitative interviews were conducted to gather data on barriers and facilitators in preventing PTB and implementing TVU in clinical practice. Select constructs from the CFIR domains were adopted for designing the semistructured interview questions. Providers were also asked to discuss potential strategies for PTB prevention and increasing TVU implementation on the patient, clinician, and system levels. Interviews were digitally recorded, professionally transcribed, and lasted ∼30–60 minutes.

Providers' attitude and knowledge related to PTB and TVU were collected through survey questions asked as part of a larger interview. Survey responses were entered into REDCap, a secure web platform for managing and creating online databases.^30^

Prenatal care records of patients seeking care at two of the community-based study sites from July 2018 to June 2019 were reviewed to evaluate the current utilization of TVU cervical length measurement for PTB prevention. Prenatal records from the third community-based site were not available for review. Data abstracted from the prenatal records were entered into REDCap.

### Data analysis

The research team analyzed interview data using a multistage coding process rooted in grounded theory^31^ using QSR NVIVO 12.^32^ Two independent coders, with oversight provided by the senior qualitative researcher, coded each transcript line-by-line and used multiple codes where appropriate to capture overlapping layered themes. Codes emerged from themes within the data.

Coders pursued several steps to enhance rigor. First, coders established a coding protocol and process to promote uniformity. Second, coders simultaneously analyzed a subsample of transcripts (representing interviews that took place at the beginning, middle, and end of the interview period), established intercoder reliability, and discussed divergences. Coders established an intercoder reliability rating of 0.80 or greater^33^ before independently coding transcripts.

Survey and medical record data were analyzed in REDCap.^30^ The research team calculated descriptive statistics of the study sample and report sample size, means, percentages, and standard deviations where appropriate.

## Results

Of the 16 providers identified and approached for study participation, 11 physicians, 1 physician assistant, 1 advanced practice registered nurse, and 1 certified nurse midwife completed interviews (*N* = 14). Six out of 14 participants were female. Average age was 44 years (SD = 10.5). Providers reported with a mean of 17 years in practice (SD = 12), although responses ranged widely from 1 to >35 years, with a mean of 5.8 years at their current practice (SD = 4.2) (Table 1). All identified as non-Hispanic white. All participants are referred to as “providers” to maintain confidentiality.

**Table 1.** Descriptive Statistics of Provider and Current Practice

	Mean	SD	Median	IQR	Range	Missing
Provide age in years	45	11	42	36–49	32–65	1
Total years in practice	17	12			1–35	2
Years at current practice	5.8	4.2			1–14	
No. of pregnant women seen per day	—	—	10	9–14	1–50	2
% of patient population at risk for PTB	22%	18%	10%	10–33%	5–50%	1

PTB, preterm birth.

All providers reported TVU cervical length evaluation was available as a diagnostic technology and imaging modality at their practice. Providers also confirmed they typically recommended TVU cervical length evaluation as part of obstetric care for at-risk women.

At one community-based site, 454 women received prenatal care during the study period from July 2018 to June 2019. Of these 454 patients, 310 had previously given birth. Of these 310 patients, 32 reported a history of spontaneous PTB (10.3%). Of these 32 at-risk patients with a prior PTB, 14 patients underwent TVU for cervical length evaluation, reflecting a rate of TVU uptake of 43.75% at this study site.

At another community-based site, 225 women received prenatal care during the study period from July 2018 to June 2019. Of these 225 patients, 147 had previously given birth. Of these 147 patients, 20 reported a history of PTB (13.6%). Of the 20 at-risk patients with a prior PTB, 10 patients underwent TVU for cervical length evaluation, reflecting a rate of TVU uptake of 50% at this study site.

Chart reviews revealed that patients had received prenatal ultrasounds, including a fetal anatomy ultrasound typically performed between 20 and 22 weeks of pregnancy. However, not all women received TVUs. This suboptimal level of TVU uptake is the result of health beliefs, social norms, and provider-level challenges. Our work calls for a multilevel intervention to address provider-, patient-, and system-level barriers to increase the uptake of TVU screening.

### Qualitative results

Themes from qualitative interviews involved risk factors for PTB, perceived barriers to TVU, and factors facilitating TVU use (Table 2). Providers were not asked about their recommendations for practices concerning specific risk factors (*i.e.*, obesity, smoking status), but rather about their TVU recommendations for at-risk women with a history of spontaneous PTB. Notably, providers identified sociodemographic factors, health behaviors, health beliefs, and clinical comorbidities as factors influencing risk of PTB. Patient access to transportation, lack of TVU training, patient comfort level, and patient social support were identified as the most common barriers limiting TVU uptake. Providers also identified a range of strategies facilitating TVU uptake, ranging from small-scale changes within their sites of practice to public service announcements concerning PTB awareness (Table 3).

**Table 2.** Themes Derived from Provider Interviews

Theme	Description	Example
		Risk factors for PTB
Sociodemographic status	Appalachia is an economically depressed region, and this was reflected in provider discussion of their patients' low socioeconomic status and widespread use of public insurance (*i.e.*, Medicaid). Providers specifically discussed how low educational attainment specifically impacted health literacy and subsequent patient/provider interaction. Another notable sociodemographic characteristic was younger average age of patients, which providers associated with a limited ability to ask questions concerning their care.	“A lot of our practice is definitely on the younger side…They just don't know that much to ask. Obviously going to school, you start reading about something, it raises more questions. The less you know, the less you can ask about it.” (Provider 8)
Health behaviors and comorbidities	Providers reported that negative health behaviors were common among their patients. The most commonly reported was tobacco use, specifically smoking cigarettes. Providers also frequently noted the high prevalence of substance abuse among their patient population as a risk for PTB, specifically opioid and illicit drug abuse and medication-assisted treatment for opioid use disorder. The most commonly mentioned comorbidity was obesity, and this aligns with the high prevalence of obesity in Appalachia. Many providers also cited high rates of diabetes.	“Then, we have two big things that really hurt us. One is obesity and one is smoking. We got a lot of smokers. Of just screening first time moms sort of thing with no history of preterm delivery, smoking is the one that's usually the biggest. That's the one that comes up the most… just for preterm delivery risk is smoking.” (Provider 10) “Our drug addicted mothers are, we are in the heart of the opioid addiction problem in the country, more so than I was when I was in central Kentucky…I don't have as much, but one of my partners sees a tremendous amount of patients that are on maintenance, in maintenance programs. I would say probably a third of his patients are in maintenance programs.” (Provider 11)
Health beliefs	The prevalence of negative health behaviors and comorbidities dovetailed with provider discussions of patients' health beliefs. According to providers, patients routinely normalized their negative behaviors (*e.g.*, smoking and illicit drug use during pregnancy) and health conditions (*e.g.*, obesity). Providers also reported that preterm birth may be normalized among their patient population, and patients frequently did not recognize the totality of risk associated with prematurity.	“…People gain weight for the same reason they always gained weight, it's just more acceptable now maybe. I don't know…I mean, it's hard to even have that discussion with people if their family's there because they just come to their defense sometimes…People don't even think of it as being unhealthy anymore because maybe it's more the norm.” (Provider 10) “And so sometimes having a preterm birth is not as scary to people. So you know like, “Well I was born at 34 weeks and I'm fine. So 34 weeks is a good time to have a baby”. And not really comprehending the long-term effects of that. So people not really taking seriously the mortality and morbidity, especially long-term effects that can happen from preterm births.” (Provider 3)
		Barriers to TVU use
	Providers identified patient access to transportation, lack of TVU training, patient comfort level, and patient social support as the most common barriers. Providers noted the necessity of a reliable care in areas with significant distances to travel lacking in public transportation. Providers conveyed patients commonly maintained an extended network of relationships with family members. However, these relationships often did not translate to tangible benefits, which may help patients overcome barriers to receiving TVU. Family members were faced with the same challenges and lacking resources as the patients.	“[I've had…conversations with patients about transportation that I'd never even thought of, and again…[in] eastern Kentucky. ‘Yeah, I don't have a car. Oh, we have a friend who drives us. We give them gas money’…We have Medicab here that works, but you have to schedule them and things like that. There are definitely attempts here to do things, but it's still hard.” (Provider 8) “Usually if they're refusing a transvaginal ultrasound, it's probably because of discomfort. So potentially if they have like a history of some sort of sexual trauma, they might not want a vaginal ultrasound or if they just are frustrated and don't want to put up with the discomfort from the procedure.” (Provider 4)
		Facilitators of TVU use
	Providers identified a variety of strategies to facilitate the use of TVUs, ranging from small-scale changes within their scope of individual practice to macrolevel cultural shifts. Small-scale suggestions for facilitating TVU included increased training for providers and sonographers, offering TVU in more locations, and extending clinic hours. Participants stressed that increasing PTB awareness and TVU-related education for patients should ideally begin early in pregnancy, and would necessitate changing community perceptions regarding consequences of PTB.	“I mean, you can always think about public service announcements, like ad campaign that make preterm birth an issue. Cause I think a lot of people maybe they don't see it as much of an issue or they say, “Oh well, my mom had me at 35 weeks and I'm fine.” So I think the social support, the lack of social support comes from that attitude. Like, “Oh, my friend's baby was fine, or my mom's baby was fine.” And I think if people were just more aware that it needed to be … that preterm birth is a big deal and is abnormal, then maybe they'd have more help from their social circles and getting to those appointments and making it a big deal.” (Provider 4) “…Formally addressing patients and screening for the risk factors and then using that as a springboard for identifying the patient, but also educating the patient that this is something that we're looking at, and this is an intervention that we can do that can help mitigate your risk factors.” (Provider 7)

TVU, transvaginal ultrasound.

**Table 3.** Provider-Reported Barriers to Transvaginal Ultrasound Use

	Median	IQR	Range	N missing
Lack of training	1	1–3	1–5	0
Lack of equipment	1	1–2	1–4	0
Equipment malfunction	1	1–1	1–3	0
Lack of equipment maintenance	1	1–2	1–4	0
Patient's insurance coverage	1	1–2	1–4	1
Provider's time	1	1–1	1–4	1
Technology or sonographer's time	1	1–2	1–4	3
Patient's time	1.5	1–2	1–4	0
Cost to patient	1	1–1	1–4	0
Cost to practice	1	1–1	1–2	1
Patient's access to reliable transportation	4	4–4	2–5	0
Patient's comfort level	3	1–3	1–4	1
Technology or sonographer's comfort level	1	1–1	1–3	4
Patient's social support	1	1–3	1–5	0

## Discussion

The current standard of care in patients with a prior PTB begins with TVU surveillance of cervical length.^34^ The present study focused on understanding Appalachian providers' knowledge, attitudes, and perceptions toward TVU surveillance, and identifying key barriers limiting widespread uptake of this evidence-based practice. The overall goal of this project is to design and pilot a tailored multilevel intervention to improve TVU cervical length surveillance among pregnant women in Appalachia with a history of PTB. This intervention will ultimately facilitate the identification of women at the highest risk for recurrent PTB and the initiation of appropriate clinical therapy.

Providers widely accepted the importance of TVU in the detection of shortened cervical length, and confirmed the availability of TVU at their practice locations. Moreover, all providers interviewed in this study reported they typically recommended TVU cervical length evaluation as part of obstetric care for their at-risk patients. However, quantitative analysis of patient data at the study sites revealed a significant gap in health care delivery, in which a fraction of women with a history of PTB received a documented TVU for cervical length measurement during their current pregnancy. This represents a critical *outcome gap* in implementation science and illustrates the need for effective translation of evidence-based medicine recommendations into clinical practice.^35,36^

The proportion of patients with documented TVUs varied between practice sites. This study is unable to report individual provider-level data regarding TVU utilization since the providers in these community-based practices typically share patient care within their group. A patient may potentially be seen by a different provider at each visit throughout her pregnancy on an alternating basis. While it is certainly possible that provider-level factors contribute to the care gap, provider interviews shed light on numerous factors, including patients' health behaviors, systemic barriers, and sociocultural norms, that likely influence TVU uptake in Appalachian Kentucky.

Providers acknowledged a distinct risk profile among their Appalachian patients reflective of sociodemographic characteristics, detrimental health behaviors, and chronic prepregnancy heath issues. All providers reported that the majority of their patients were of low socioeconomic status (SES), reflected in their use of public health insurance and lower levels of health literacy. Closely intertwined with sociodemographic characteristics were health behaviors providers directly associated with increased PTB risk. Namely, providers identified a high prevalence of smoking among pregnant women as a direct contributor to PTB, and stated smoking was typically normalized among their patient population. Providers also identified a high prevalence of substance abuse, specifically opioid use disorder and the need for medication-assisted treatment (opioid maintenance therapy), among their patients. Providers associated substance abuse with challenges to prenatal care adherence. Lastly, providers noted widespread prepregnancy obesity among their patients and a recent tendency toward normalization of obesity among pregnant women. Providers noted chronic conditions alongside obesity, such as hypertension and diabetes, further complicated prenatal care and PTB risk. Multiple providers acknowledged that although this risk profile increased women's likelihood of experiencing a PTB, the commonplace nature of such health conditions and behaviors contributed to a deemphasizing of the dangers of PTB.

Providers additionally identified multiple systemic barriers that complicated TVU use. Patient transportation was repeatedly identified by providers as a barrier to TVU, and more generally, to prenatal care access. Transportation difficulties were attributed to long transit times required by an additional clinic visit, as well as unreliable means of transportation among low-SES patients. Providers also identified patient comfort level and patient social support as potential barriers to TVU use, but commented that patient discomfort rarely resulted in refusal of TVU. Providers noted that pregnant women were largely willing to undergo procedural care, although such factors have been significant enough for patients to deny a TVU in nonobstetric gynecologic patients. Regardless of whether or not patient discomfort resulted in TVU refusal, many providers recognized that patients with a history of sexual trauma, and adolescent and teen patients may be uncomfortable with TVU use.

The most deeply seeded barriers to TVU were founded in the sociocultural norms of the patient population concerning PTB. Providers expressed that patients commonly adopted the experiential and anecdotal knowledge of their community members rather than the evidence-based knowledge imparted by the provider. The knowledge of other women in the community, especially maternal figures within the family, may be prioritized over information provided by the practitioner. Specifically, providers stated that patients commonly perceived PTB as unproblematic because they had witnessed other mothers in their community experience a PTB with no obvious consequences. From the perspective of providers, the totality of risks associated with PTB was not widely understood by patients. Throughout the community, providers noted PTB was frequently not perceived as detrimental or associated with long-term consequences to the infant. In sum, providers identified barriers to prenatal care and risk factors for preterm labor, which may manifest in this population as decreased screening.

Providers further identified potential mechanisms for the facilitation of TVU use. Multiple providers commented that more reliable means of transportation may aid in TVU use. Providers noted that transportation options are limited in rural areas, and many patients cannot afford a for-hire mode of transportation. However, providers mentioned clinics may provide a form of transportation for patients requiring care. Other providers expressed that changing community perceptions regarding consequences of PTB and enhancing patient awareness of TVU use may encourage women to initiate PTB screening and seek services earlier in pregnancy. Restructuring the communities' awareness of PTB risk may shift patients understanding so that PTB is no longer as normalized. Lastly, providers noted that implementing TVU use within the prenatal care clinic as a point-of-care service may alleviate the logistical difficulty associated with additional clinic visits.

Lack of TVU training was largely reported as a barrier by providers without primary specialty training in obstetrics and gynecology. While ultrasound services were noted to be available at all community-based sites, the actual accession route varied from one community clinic to another. TVUs are either performed in the office setting by sonographers (with scans read remotely by maternal fetal medicine specialists), or performed by radiology service providers (with scans read by radiologists). At no location were ultrasounds readily available at the community clinic. Lack of training may therefore refer to providers' inability to both perform TVUs on site and reliably read ultrasounds. The inability to perform services on site may also exacerbate other challenges to TVU uptake attributable to additional clinic visits, such as challenges surrounding patient transportation and constraints on patients' time. Providers consistently identified TVU cervical length surveillance as important for the assessment of PTB risk, and voiced a willingness for future TVU education. Past provider-level interventions have demonstrated that a structured educational module improves providers' ability to obtain a high-quality image and accurately measure cervical length *via* TVU.^37,38^ The openness of providers to both in-person and online modules indicates that the development of an educational intervention may improve TVU utilization and imaging competence.

The identification of distinct barriers and facilitators in Appalachian Kentucky is necessary to alleviate the region's high rate of PTB. Patients in Appalachian Kentucky face limitations posed by access to care, transportation, lack of health literacy, and resources required for increased frequency of health care visits. Unlike the predominantly urban or suburban areas of the country, prenatal care providers in Appalachian Kentucky represent a blend of physicians (*e.g.*, obstetrics and gynecology, family medicine) as well as auxiliary providers including nurse practitioners and nurse midwives—thus encompassing a wide spectrum of clinical knowledge, practice experiences, and perceptions that further enrich study findings. These findings are a critical first step in designing a targeted intervention for PTB prevention in Appalachian Kentucky with an emphasis on increasing uptake of TVU cervical length screening to identify at-risk patients and initiate appropriate medical or surgical therapy.

Although there exists a strong evidence base in support of universal cervical length screening,^17,39^ there is limited research into barriers preventing implementation. Past studies examining implementation of cervical length screening have focused on providers associated with large, academic medical centers in urban settings.^13–15,40^ In contrast, providers included in this study practiced in community clinics in largely rural areas. Circumstances faced by providers in rural Appalachian Kentucky are distinct from providers in urban academic centers. This study importantly elucidates barriers to implementation of cervical length screening in an understudied population.

Qualitative methods used in this study allow for an in-depth evaluation of providers' experiences concerning PTB and TVU use in Appalachia. Interviews with providers produce rich, highly contextualized data concerning complex barriers and facilitators impacting patients' risk for PTB. Prenatal care in Appalachian Kentucky involves a combination of professionals with varying levels of training and diverse skill sets, including physicians as well as auxiliary providers including nurse practitioners and nurse midwives. Inclusion of these diverse professional perspectives within this study allows for a more comprehensive understanding of patient management, provider/patient interactions, and uptake of evidence-based practices. However, the methods used in this study limit the sample size and generalizability of results. This study was designed as a pilot and limited to three community-based clinics in Appalachia.

Future directions of this study will address limited generalizability of findings by expanding to other community clinics in Appalachia and involving other stakeholders (*e.g.*, clinic leadership/administrators, sonographers, nurses). Results of this study will help inform future efforts implementing comprehensive cervical length screening in an underserved rural population, and potentially other similarly isolated and health disparate populations, thus serving as a model for dissemination.

## Abbreviations Used

CFIR: Consolidated Framework for Implementation Research
PTB: preterm birth
SES: socioeconomic status
TVU: transvaginal ultrasound
